# Is There an Association between Sleeping Patterns and Other Environmental Factors with Obesity and Blood Pressure in an Urban African Population?

**DOI:** 10.1371/journal.pone.0131081

**Published:** 2015-10-08

**Authors:** Sandra Pretorius, Simon Stewart, Melinda J. Carrington, Kim Lamont, Karen Sliwa, Nigel J. Crowther

**Affiliations:** 1 Soweto Cardiovascular Research Unit, University of the Witwatersrand, Johannesburg, South Africa; 2 Preventative Cardiology/National Health and Medical Research Council of Australia Centre of Research Excellence to Reduce Inequality in Heart Disease, Baker IDI Heart and Diabetes Institute, Melbourne, Australia; 3 Hatter Cardiovascular Research Institute, Department of Medicine, Faculty of Health Sciences, University of Cape Town, Cape Town, South Africa; 4 Department of Chemical Pathology, National Health Laboratory Service, University of the Witwatersrand, Johannesburg, South Africa; Vanderbilt University, UNITED STATES

## Abstract

Beyond changing dietary patterns, there is a paucity of data to fully explain the high prevalence of obesity and hypertension in urban African populations. The aim of this study was to determine whether other environmental factors (including sleep duration, smoking and physical activity) are related to body anthropometry and blood pressure (BP). Data were collected on 1311 subjects, attending two primary health care clinics in Soweto, South Africa. Questionnaires were used to obtain data on education, employment, exercise, smoking and sleep duration. Anthropometric and BP measurements were taken. Subjects comprised 862 women (mean age 41 ± 16 years and mean BMI 29.9 ± 9.2 kg/m^2^) and 449 men (38 ± 14 years and 24.8 ± 8.3 kg/m^2^). In females, ANOVA showed that former smokers had a higher BMI (p<0.001) than current smokers, while exposure to second hand smoking was associated with a lower BMI (p<0.001) in both genders. Regression analyses demonstrated that longer sleep duration was associated with a lower BMI (p<0.05) in older females only, and not in males, whilst in males napping during the day for > 30 minutes was related to a lower BMI (β = -0.04, p<0.01) and waist circumference (β = -0.03, p<0.001). Within males, napping for >30 minutes/day was related to lower systolic (β = -0.02, p<0.05) and lower diastolic BP (β = -0.02, p = 0.05). Longer night time sleep duration was associated with higher diastolic (β = 0.005, p<0.01) and systolic BP (β = 0.003, p<0.05) in females. No health benefits were noted for physical activity. These data suggest that environmental factors rarely collected in African populations are related, in gender-specific ways, to body anthropometry and blood pressure. Further research is required to fully elucidate these associations and how they might be translated into public health programs to combat high levels of obesity and hypertension.

## Introduction

Previous studies performed in an urban African population living in Soweto, South Africa have shown prevalences for obesity of 42–50% in women [1,2]. Income inequalities in South Africa, as in the rest of Africa, has resulted in populations migrating from rural to urban areas, leading to changes in lifestyle and a higher prevalence of obesity, which has become, due to its associated diseases, a major public health concern [2,3]. The increasing prevalence of obesity and its comorbid diseases require a better understanding of the contributing factors that predispose individuals to gain weight. This is necessary to plan intervention strategies and thus improve public health [4].

An association between weight gain, obesity and sleep duration has consistently been demonstrated in recent research. It has been shown that sleep duration can have important effects on health and that both short (<7 hours sleep a night) or long sleep duration (≥ 9 hours sleep a night) may represent a risk marker for poorer health outcomes rather than a causal risk factor for diseases [5]. Several studies have reported a relationship between sleep duration and chronic diseases, such as heart disease, diabetes and obesity [6,7,8]. Longer sleep has been associated with unemployment, low education levels, low income, alcohol consumption, depression, low physical activity levels, pregnancy and ethnicity. Short sleep duration is associated with weight gain and obesity, diabetes mellitus, cardiovascular disease, psychiatric illness, performance deficits, as well as higher levels of schooling and income, longer working hours and being single [4,8,9].

The prevalence of obesity within a population is also known to be associated with social factors such as urbanisation [10,11], education and socio-economic status (SES) [12]. Meta-analyses of the relevant literature have shown that in developed countries a negative relationship exists between SES and obesity while this moves to a positive relationship in low income nations [12,13]. Studies have also indicated that smoking cessation leads to increases in body weight [14]. Smoking and obesity are leading causes of morbidity and mortality worldwide and are associated with increased risk of cancers, high blood pressure, ischaemic heart disease and diabetes [15]. The relationship between smoking and obesity is complex. The fact that smokers have a lower body mass index (BMI) than non-smokers can be explained by the effect of nicotine, which increases energy expenditure and reduces appetite [15,16]. Furthermore, smoking cessation is frequently followed by weight gain [14,15]. However, studies indicate that heavy smokers have a higher BMI than light smokers (8–10 cigarettes per day) [15,16]. It is therefore important that variables, such as smoking history should also be quantified when analysing risk factors for obesity in different population groups.

Studies linking sleep duration and smoking status to obesity and related metabolic diseases have largely been restricted to populations in developed countries, with very little data available from African nations. The social determinants of obesity have also been under studied in these countries. Developing nations in Africa have a rising prevalence of obesity, particularly in urban, female populations [17,18]. Therefore, the aim of the current study was to assess the relationship of social factors, smoking status and sleep duration with measures of obesity in an urban African population resident in the Johannesburg-Soweto conurbation.

## Methods

### Study setting

The overall goal of the Heart of Soweto study (HOS) was to systematically examine and respond to the epidemiologic transition in risk behaviours and clinical presentations of heart disease in an urban African community in Soweto, South Africa [19]. In order to assess chronic diseases of lifestyle, such as heart disease, diabetes, high blood pressure and obesity, and to plan appropriate intervention strategies, we extended our research into the Soweto community and primary health care setting [1]. Consistent with the HOS clinical registry [19] data were systematically collected on consecutive patients attending two pre-selected primary care clinics from a total of 12 clinics in Soweto (644 and 667 patients from Mandela Sisulu, Orlando West and Michael Maponya, Pimville primary health care clinics in Soweto, respectively). These two primary health care clinics were chosen as they are situated in two diverse socio-economic locations in Soweto. The study was undertaken over a 6 month period and involving 50 discrete days of screening (commencing June 2006) [20].

### Participants

Each primary care clinic typically manages more than 300 patients per day with wide-ranging health issues. A study team comprising an experienced cardiac nurse, ECG technician and co-ordinator invited consecutive consenting patients aged over 16 years who presented to the primary care clinic to be screened. All patients were reviewed by a primary health care nurse prior to assessment. A target of assessing approximately 25 consecutive patients each screening day was maintained during the study period [20].

### Study data collection

Each participant was subject to a standardised program of assessment as follows (information was collected from patients using a questionnaire that was used in a previous study within this population group) [20]: 1. Self-reported cultural and socio-demographic profile including ethnic origin (African, European, Indian or mixed ancestry), duration of residence in Soweto, highest level of education (none, junior school only, high school without graduating, graduated from high school, tertiary education) and whether currently employed; 2. Risk factor profiling, including smoking status. 3. Anthropometric profile including height and weight with calculation of BMI. Weight was measured with a calibrated Seca 767 electronic scale (Lifemax, Johannesburg, South Africa) that weighs up to 200 kg and height was measured with a Seca 220 telescopic measuring rod according to acceptable standardised methods [21]. The WHO guidelines were used to classify individuals as obese (BMI of 30 or more) [9]. Waist and hip circumference were measured with a standardised measuring tape calibrated in cm. All participants were weighed and measured with their clothes on, but without their shoes; 4. Blood pressure was measured on the right arm by a registered nurse, using the Omron automatic digital blood pressure monitor (Omron M10-IT BPM-Digital, Johannesburg, South Africa). The subject was seated upright and relaxed with his/her right arm supported at heart level. Subjects were instructed to refrain from eating, smoking, ingesting caffeine or exercise/physical activity such as climbing the stairs in the 30 minutes prior to the measurement. HT was diagnosed i.e. subjects with BPs of 140/90 according to South African Hypertension Society guidelines and/or those who were being treated for HT [1,22]; 5. Medical history and management including prior or current diagnoses of diabetes and hypertension and pharmacological therapy related to the treatment of hypertension; 6. Self-reported sleep duration was assessed by asking participants the following questions: “During your longest or nocturnal sleep period, what time do you normally go to bed?” and “During your longest or nocturnal sleep period, what time do you normally wake up?”; 7. Self-reported napping was assessed by asking participants the following questions: “Do you usually take a nap, yes or no?” and “If yes, total nap duration in minutes?”. 8. Smoke exposure was measured by asking participants the following questions: “During the past 12 months, have you been regularly (at least once per week) exposed to other people’s tobacco smoke and what has been your typical exposure? (“Exposed” is defined as a minimum of 5 consecutive minutes, during which you inhale other people’s smoke).

### Statistical Analysis

All statistical analyses were performed using Statistica version 12 SP2 (StatSoft, Tulsa, OK, USA). Normally distributed continuous data are presented as the mean ± standard deviation. Data that was not normally distributed are presented as the median (interquartile range (IQR)) and these variables were log transformed to normality before being analysed using parametric statistical tests.

Means for continuous variables were compared between 2 groups using Students t test, whilst trends across 3 or more groups were assessed using ANOVA with the Tukey HSD post hoc test used for the comparison of paired means. Percentage values were compared across groups using the χ^2^ test. Multiple linear regression models were developed to identify the determinants of anthropometric and metabolic variables. The independent variables included in the initial regression models were chosen based on previous statistical analyses and biological plausibility. The independent variables included in the models for BMI were: age, education level, employment status, smoking status, exposure to tobacco smoke, night time sleep duration, day time nap duration, walking distance per day, diabetes, hypertension and treatment type, HIV and TB status. The regression models for waist included all above variables plus BMI. The regression models for systolic and diastolic blood pressures included the same independent variables as for the BMI models but included BMI and waist but excluded hypertension. The same variables were included in the regression models for males and females. Only the independent variables that had p≤0.05 are reported in the results.

The linearity of all continuous variables was analysed by the observation of scatter plots of observed versus predicted values and residuals versus predicted values. These showed that all continuous variables were linear with the exception of sleep duration. This variable was therefore used in all regression models as either a continuous variable or as a categorical variable. The categorical variable was generated by creating dummy variables for quartiles, with the lowest quartile as the reference.

Outliers were identified by observation of normal probability plots used in combination with lower and upper cut points defined as: lower quartile boundary–(1.5 X IQR) and upper quartile boundary + (1.5 X IQR), respectively. A number of data points that fell above the upper cut point for BMI were observed. Removal of these from the dataset during statistical analysis had minimal effects on the outputs.

### Ethics Statement

The Chairman of the human Research Ethics Committee (Medical) of the University of the Witwatersrand has reviewed and approved the request to continue with the Heart of Soweto Study and extending the collection of data into primary health care clinics in Soweto, protocol number, M050550.

As with the ‘Heart of Soweto Registry’, of which data from the first 4162 cases has been published in ‘The Lancet’ (2008) [19], every patient in the survey was assigned a unique identifying code (nine digits), and all documentation were labelled accordingly to maintain anonymity.

All participating patients provided verbal consent to become part of the survey, as this was in line with registries at the University of the Witwatersrand at the time, as approved by the ethics committee. Only patients who gave verbal consent were recruited into the survey and a registration form was then completed for those patients who consented.

## Results

### Characteristics of Study Population

A total of 1311 adult subjects were interviewed, comprising 862 women (66% of the study group) and 449 men and of whom 1294 classified themselves as black African. As summarised in Table 1 females were approximately 3.5 years older than males and had a nearly 3-fold higher prevalence of obesity and had median waist and hip circumferences that were 9.0 and 12.0 cm greater than for males (p<0.001 for all comparisons). Sleep duration (p<0.01) was 0.30 hours higher in females. Nearly 4-times as many men as women were current smokers (p<0.001) and the frequency of the exposure to second hand cigarette smoke was 8.2% higher in men (p<0.01). The level of unemployment was 12.0% higher in males than females (p<0.001). No gender differences were noted for either diastolic or systolic blood pressure levels however, the prevalence of hypertension was 12.5% higher in females than males (p<0.001), and within the total cohort 52.5% of hypertensive subjects were receiving therapy for hypertension. The anti-hypertensive agents being used and their frequency of use within the hypertensive subjects were: thiazide diuretics (48.1%), ACE inhibitors (22.9%), nifedipine (17.8%), beta blockers (3.2%), furosemide (2.4%) and spironolactone (0.48%). Within the hypertensive subjects, 61.5% were receiving 2 or more anti-hypertensive agents.

**Table 1:** Comparison of male and female subjects.

**Variables**	Males	Females	Combined
*Age (years)*	37.6 ± 14.0	41.1 ± 16.4***	39.9 ± 15.7
*BMI*	22.9 (20.5, 26.7)	28.3 (23.5, 34.7)***	26.1 (22.1, 32.4)
*Obesity (%)*	14.1	41.8***	32.4
*Waist (cm)*	83.0 (76.0, 92.0)	92.0 (81.0, 103)***	89.0 (78.0, 100)
*Hip (cm)*	98.0 (91.0, 105)	110 (100, 121)***	105 (96.0, 117)
*Diastolic bp (mmHg)*	81.3 (74.0, 91.0)	83.0 (74.3, 93.0)	82.7 (74.3, 92.3)
*Systolic bp (mmHg)*	128 (117, 142)	129 (116, 145)	129 (117, 144)
*Hypertension (%)*	39.9	52.5***	48.2
*Sleep duration (hours)*	8.55 ± 1.78	8.85 ± 1.67**	8.75 ± 1.72
*Sleep in the day (%)*	28.3	29.6	29.2
*Current smoker (%)*	36.2	9.62***	18.6
*Smoke exposed (%)*	80.6	72.4**	75.2
*High school graduates (%)*	45.5	41.5	42.9
*Employed (%)*	37.8	25.8***	29.8
*Walk > 2km/day (%)*	75.7	71.8	73.1
*HIV-positive (%)*	5.69	6.68	6.35
*TB-positive (%)*	2.96	2.46	2.63

Age and sleep duration are expressed as mean ± SD, whilst BMI, waist, hip, diastolic and systolic blood pressure are given as median (interquartile range) and the remaining variables are expressed as percentages

**p<0.01

***p<0.001 versus males.

### Anthropometric effects of sleep, smoking, education, employment, exercise, HIV and TB

Table 2 shows that sleeping during the day (napping) was associated with a lower BMI in males but this effect was not significant in females. In both genders, current smokers had lower BMIs than those who never smoked, whilst in females former smokers had a significantly higher BMI than current smokers. Exposure to second hand cigarette smoke was associated with a significantly lower BMI in both genders. Subjects who graduated from high school had lower BMIs than those who did not graduate, and this effect was significant in males and females. Neither employment status nor walking more than 2 km per day had a significant effect on BMI in either gender. Within females but not males, HIV and TB positivity were both associated with a significantly lower BMI than that observed in subjects without these infections.

**Table 2:** BMI values in different population sub-groups for each gender.

Variables		Males			Females		
		BMI	β-value (%)	n	BMI	β-value (%)	n
***Sleep in the day***	***No***	23.2 (21.0, 26.9)	-	296	28.6 (23.5, 35.0)	-	569
	***Yes***	22.4 (19.9, 25.3)*	-2.49±1.17	116	27.2 (23.1, 33.6)	-1.58±0.92	237
***Smoking status***	***Current***	21.9 (19.6, 24.1)	-	157	24.9 (21.5, 29.7)	-	82
	***Former***	22.3 (20.5, 25.4)	1.45±1.77	46	30.7 (25.2, 36.3)***	7.81±2.25	40
	***Never***	24.2 (21.5, 27.7)***	5.34±1.10	226	28.6 (23.6, 34.7)***	5.76±1.36	711
***Smoke exposed***	***No***	24.5 (21.3, 30.6)	-	79	30.7 (26.1, 36.3)	-	225
	***Yes***	22.8 (20.3, 26.1)***	-4.86±1.33	347	27.2 (22.8, 33.3)***	-4.36±4.79	607
***High school graduate***	***No***	23.3 (21.2, 27.3)	-	232	29.8 (24.8, 36.0)	-	485
	***Yes***	22.2 (20.1, 26.2)*	-2.00±1.05	195	26.3 (22.2, 32.0)***	-4.46±0.82	341
***Employed***	***No***	23.2 (21.0, 27.0)	-	242	28.3 (23.7, 35.0)	-	591
	***Yes***	22.5 920.6, 26.0)	-1.55±1.14	149	28.6 (23.6, 34.4)	0.05±0.96	207
***Walk > 2km/day***	***No***	23.5 (21.1, 27.6)	-	101	30.0 (23.1, 35.0)	-	233
	***Yes***	22.8 (20.3, 26.2)	-1.84±1.23	319	27.7 (23.6, 34.4)	-1.43±0.91	593
***HIV status***	***Negative***	22.8 (20.5, 26.5)	-	407	28.6 (23.6, 35.0)	-	777
	***Positive***	24.7 (20.3, 29.9)*	5.48±2.26	24	25.5 (22.8, 30.7)*	-4.46±1.61	57
***TB status***	***Negative***	22.9 (20.6, 26.6)	-	418	28.4 (23.5, 34.7)	-	813
	***Positive***	20.8 (19.0, 28.6)	4.74±3.04	13	24.8 (21.9, 27.3)**	-7.45±2.60	21

Data is expressed as median (interquartile range)

*p≤0.05

**p<0.01

***p<0.001

BMI was logged; β-values (effect sizes) were multiplied by 100 to give percentage values (± SD) and were generated from regression models with “No” or “Negative” coded as 0 and “Yes” or “Positive” coded as 1 and for smoking status, “Former” and “Never” were both compared against”Current”.

Waist circumference was also analysed across the same exposure groups as depicted in Table 2. Smoking status, smoke exposure, education and HIV status all had the same effects on waist circumference (data not shown) as those observed for BMI (see Table 2). In males, sleep during the day had a similar effect on waist circumference as for BMI, whilst in females BMI was not significantly affected (see Table 2) but waist circumference was lower in those who slept during the day (90.0 [78.0, 102] vs. 92.0 [82.0, 104]; p<0.05). In males but not females, subjects who were employed had a lower waist circumference than those not employed (82.0 [75.5, 90.0] vs. 84.0 [76.0, 94.0]; p<0.05). Walking more than 2 km per day was associated with a lower waist circumference in females (91.0 [80.0, 102] vs. 95.0 [81.0, 108]; p<0.05), but not males. In females who were TB-positive, both BMI and waist circumference were significantly lower than in uninfected subjects whilst in males BMI was not affected (see Table 2) but waist circumference was lower in TB-positive compared to TB-negative men (74.0 [69.0, 80.0] vs. 83.0 [76.0, 92.0]; p<0.05).

### Effect of smoke exposure on BMI and waist circumference

The data in S1 Fig shows that in males (ANOVA, p<0.001) and females (ANOVA, p<0.001) BMI falls with increasing exposure to second hand cigarette smoke. The analysis in these 2 groups includes subjects who are current, former and non-smokers. If the comparison is only performed in non-smokers, the effect is still statistically significant (ANOVA, p<0.001) with the highest BMI (29.3 [25.0, 35.3]) observed in subjects not exposed to environmental tobacco smoke, and the lowest BMI (25.5 [21.7, 31.8]) in subjects exposed to tobacco smoke > 3 times/day. Similar trends were observed for waist circumference in males (p<0.05), females (p<0.001), and non-smokers (p<0.001).

### Determinants of BMI and waist circumference in males and females

The results of linear multiple regression models for the identification of the determinants of body anthropometry and blood pressure are shown in Table 3. Age is shown to correlate positively and strongly (p<0.001) with all the dependent variables, with the exception of waist circumference in females.

**Table 3:** Multiple regression models for BMI, waist circumference and blood pressure in male and female subjects.

Model no. & gender	Dependent variable	Independent variables with unstandardized B (p-value)	Whole model R^2^ (p-value)
		Age 0.001 (0.02)	0.13 (<0.001)
		Smoking -0.03 (0.008)	
***1—male***	BMI	Nap >30 mins -0.04 (0.006)	
		Treated HT 0.04 (0.02)	
		Age 0.002(<0.001)	
		Smoking -0.04 (0.004)	0.17 (<0.001)
***2—female***	BMI	Smoke exposure -0.02 (0.03)	
		Non-hypertensive -0.02 (0.04)	
		Age 0.001 (<0.001)	
		BMI 0.27(<0.001)	
		Smoking -0.01 (0.03)	
***3—male***	Waist	Nap >30 mins -0.03 (<0.001)	0.38 (<0.001)
		Diabetes 0.05 (0.01)	
		HIV -0.04 (0.005)	
		TB -0.06 (0.01)	
***4—female***	Waist	BMI 0.42 (<0.001)	0.48 (<0.001)
		Age 0.001 (<0.001)	
***5- male***	Systolic bp	Waist 0.18 (0.002)	0.16 (<0.001)
		Nap >30 mins -0.02 (0.01)	
		Age 0.001 (<0.001)	
***6—female***	Systolic bp	Waist 0.19 (<0.001)	
		Sleep duration 0.003 (0.03)	0.22 (<0.001)
		Age 0.001 (<0.001)	
		Waist 0.14 (0.03)	
***7—male***	Diastolic bp	Nap >30 mins -0.02 (0.05)	0.11 (<0.001)
		Age 0.0008 (<0.001)	
		Waist 0.21 (<0.001)	
		Education -0.01 (0.03)	
***8—female***	Diastolic bp	Nap>30 mins -0.02 (0.05)	0.16 (<0.001)
		Sleep duration 0.005 (0.002)	
		Walking 0.01 (0.05)	

The variables BMI, waist, systolic and diastolic blood pressure are all logged. Variable coding: Smoking, smoke exposure and employment–yes = 1, no = 0; TB and HIV–positive = 1, negative = 0; education—graduated from high school = 1, did not graduate = 0; walking—walk > 2km/day = 1, < 2km/day = 0; napping during the day was coded using dummy variables with subjects who did not nap used as the reference group and compared with subjects who napped ≤ 30 minutes/day and subjects who napped > 30 minutes/day; hypertension (HT) was coded using dummy variables with non-treated hypertensives used as the reference group and compared with non-hypertensives and treated hypertensives

Subjects who smoke have a BMI that is 4.0% lower in females and 3% lower in males than those who do not smoke. Furthermore, subjects who are environmentally exposed to tobacco smoke have a BMI that is 2.0% lower in both males and females than those who are not exposed, but this association was significant only in females (p = 0.03; p = 0.14 in males). In males, subjects who sleep during the day for more than 30 minutes have a BMI that is 4.0% lower than those who do not sleep in the day. In model 2 for females, a negative but non-significant association (β = -0.004; p = 0.14) was observed between sleep duration and BMI, whereas in a univariate analysis a significant association was observed (β = -0.007; p = 0.005). When age was removed from the multivariable model (model 2 in Table 3) the association between BMI and sleep duration became significant (β = -0.005; p = 0.04). Further analysis demonstrated that in a multivariable regression model for BMI that included age, sleep duration and an interaction term (age X sleep duration), the interaction term was negative and significant (β = -0.0003; p = 0.02), This interaction was confirmed by running univariate regression models for females above and below the median age (40 years). In the model for females below age 40, no significant association between BMI and sleep duration was noted (β = -0.0003; p = 0.93), but a significant relationship was noted in females with age above the median (β = -0.008; p = 0.02). These relationships are depicted in S2 Fig, where BMI falls significantly across quartiles of sleep duration in females with age ≥ 40 years (ANOVA, p<0.05) but not in females < 40 years (ANOVA, p = 0.88). No such interaction was observed for male subjects. In males receiving treatment for hypertension BMI is 4.0% higher than hypertensive males who are not receiving therapy. In females without hypertension, BMI is 2.0% lower than in hypertensive females not receiving therapy.

Males who smoke have a waist circumference that is 1.0% lower than those who do not smoke. Male subjects who sleep for more than 30 minutes during the day have a waist circumference that is 3.0% lower than those who do not sleep in the day. Diabetic male subjects have a waist circumference that is 5.0% higher than non-diabetic subjects. Males who have HIV or TB infections have a waist circumference that is 4.0% or 6.0% lower respectively, than non-infected subjects. In non-hypertensive females, waist circumference is 2.0% lower than in hypertensive females not receiving therapy.

The data in Table 2 showed that waist circumference was lower in females who walked more than 2km per day compared to those who did not. However this relationship was not observed in the multiple regression analysis depicted in model 4 of Table 3 suggesting that the significant effect of exercise observed in Table 2 is the result of confounding.

### Determinants of blood pressure in males and females

Systolic and diastolic blood pressure correlate significantly with waist circumference in both genders (Table 3). Within females, diastolic blood pressure is 1.0% lower in subjects who graduated from high school when compared to those who did not graduate. Diastolic blood pressure in females is 1.0% higher in those who walk more than 2 km per day when compared to those who walk less than this distance. In males, systolic and diastolic blood pressure are both 2.0% lower in those who sleep for more than 30 minutes during the day and diastolic blood pressure is also 2.0% lower in females who sleep more than 30 minutes in the day when compared to subjects who do not sleep in the day. Systolic blood pressure is 0.30% lower and diastolic 0.50% lower for every extra hour of night time sleep in female subjects. Due to the non-linearity of the relationship between sleep duration and blood pressure, sleep duration was divided into quartiles and dummy variables generated with the first quartile used as the reference. This was done only for the female subjects. These dummy variables were then included in models 6 and 8 as replacements for the continuous sleep duration variable. The median blood pressure levels were also calculated for each sleep duration quartile. Diastolic blood pressure rose from 81.8 (73.0, 91.0) in quartile 1 to 85.0 (75.7, 92.3) in quartile 2, 82.7 (75.3, 95.3) in quartile 3 and 83.0 (75.0, 93.3) mmHg in quartile 4 (p<0.05 for each of the top 3 quartiles versus quartile 1). This trend was repeated for systolic blood pressure. The replacement of the continuous sleep duration variable with a categorical variable did not affect the outputs of the other independent variables for either the diastolic or systolic regression models. An interaction term of age X sleep duration was also generated but showed no significant relationship with either systolic or diastolic blood pressure in the female subjects.

## Discussion

This study confirms previous reports demonstrating a much higher prevalence of obesity in urban dwelling African women versus men [1,2]. The results showing that sleep duration in females and napping during the day in males is associated with both lower BMI and lower blood pressure is the first such data from an African study. Furthermore, this cross sectional study is the first to show that in an African population there is a negative relationship between smoking and BMI and is also the first to demonstrate that environmental tobacco smoke exposure is related to a lower body adiposity.

Female subjects in this cohort had a much higher prevalence of obesity, with 41.8% of females being obese compared to 14.1% of males. Females also had significantly higher waist and hip circumferences when compared to males. These results confirm data from previous studies performed in Soweto, showing that urban African females have a very high prevalence of obesity [1,2]. The much higher prevalence of obesity in females than males is characteristic of African populations [17,18], the reason for which is largely unknown.

The present study is the first to confirm the strong inverse effect of smoking on levels of obesity in an indigenous African population. Thus, BMI was higher in non-smokers when compared to those who currently smoke. Studies have previously shown that smoking may increase energy expenditure and reduce appetite [9,16]. Furthermore, it is known that weight gain occurs after people stop smoking and this is confirmed in the present study where subjects who have ceased smoking had higher BMIs than those who still smoke, especially amongst females [14–16]. Our data also demonstrates that male and female subjects that have been exposed to second hand cigarette smoke have a lower BMI than non-exposed subjects. This effect was stronger in females and was almost as strong as that observed for primary smokers. It was also seen in those individuals who are not smokers themselves. Previous studies have shown that passive smoke exposure during pregnancy or in infancy increase the risk of obesity in childhood [21,23] whilst the current study is the first to demonstrate that environmental tobacco smoke exposure in adults is associated with a lower BMI. Our observation from the present study suggest that national smoking intervention programmes must not only take cognisance of the effects of smoking cessation on weight gain in the primary smokers but also in the passive smokers. The prevalence of active smoking in African nations is high, particularly in males [24] and therefore it is possible that successful intervention programmes may have large effects on the prevalence of obesity in these countries. These observations are however, not aimed at policy change as we cannot draw sufficient conclusions from our cross-sectional study.

In the current study a negative relationship was observed between night time sleep duration and BMI in female subjects. A number of other studies have shown that inadequate sleep is associated with overweight and obesity [25–27]. It has been suggested that sleep restriction may lead to obesity via changes in the appetite regulating hormones leptin and ghrelin [28]. Thus, laboratory studies shows that with short sleep duration, leptin levels, a satiety signal, were reduced, while levels of ghrelin, an appetite stimulant, were increased. Furthermore studies have shown that short sleep duration does lead to increased caloric intake [29,30], and increased consumption of foods high in fat and refined carbohydrates has been observed after sleep restriction [4,9]. The relationship between BMI and sleep duration in females was only observed in older subjects. No other studies have shown a modifying effect of age on the relationship between sleep and BMI, and previous investigations have shown that the negative relationship between sleep duration and obesity risk is observed in both children and adults [31]. Therefore, this finding may be unique to our population however, more studies across different ethnic groups are required to confirm this observation.

The present study also demonstrated that female subjects with longer sleep duration had higher systolic and diastolic blood pressures. The relationship between sleep duration and blood pressure is complex, with studies showing a ‘U’ shaped association [32]. A ‘U’ shaped relationship was not observed in the present study, with both systolic and diastolic blood pressure in females initially increasing with longer sleep duration and then falling. The relationship between sleep duration and blood pressure also changes according to age group and gender with females aged 18–44 years and ≥ 65 years demonstrating a greater risk of hypertension at a sleep duration of < 6 hours, whilst females aged 45–65 years demonstrated the highest risk of hypertension at a sleep duration of ≥ 10 hours [32]. No interaction was observed in the current study between age, sleep duration and BMI in the female subjects. The reasons why blood pressure increases at high levels of sleep duration is not fully understood.

In males, no association was observed between sleep duration and BMI however, males who slept during the day had a lower BMI and a lower waist circumference than those who did not nap in the day. A previous study has also shown that sleep in the daytime (siesta) is associated with a lower BMI [33]. It has also been demonstrated that napping for 1 hour or longer is associated with an increased risk of cardiac events [34] whilst napping for 30 minutes or less has some health benefits [35]. However, the current study clearly shows that napping for 30 minutes or more reduces blood pressure in males and females. The reason for these contradictory results may be that the study showing an increased risk of cardiac events with longer nap duration used an older population (45–75 years) than used in the present study, and also used subjects of European descent [34].

Multiple regression analysis demonstrated that the effect sizes for the associations of sleep duration with BMI and blood pressure were comparatively weak (β values between 0.003 and 0.005), whilst the effects of napping during the day were considerably stronger (β values between 0.02 and 0.03). It is also interesting to note that night time sleep duration only had significant associations in females, whilst napping in the day time had more associations in males (with BMI, waist, systolic and diastolic blood pressure) than females (diastolic blood pressure). Furthermore, napping correlated negatively with blood pressure in males and females but night time sleep duration correlated positively in females. These associations do not prove causality and may be moderated by other unmeasured variables. Therefore, more in depth studies are required to investigate the differential effects of day and night time sleeping patterns on BMI and blood pressure in the different genders and to map out the possible causal relationships between these variables.

A higher level of education in female subjects was related to lower diastolic blood pressures. No such relationship was observed in men. A differential relationship of education with components of the metabolic syndrome across the genders has been observed in other investigations [36–38], but its causes are not known.

HIV and TB infection are both commonly associated with weight loss. Using regression analyses the current study demonstrates a strong relationship in males only of both HIV and TB infection with a low waist circumference. The reason for the differential effects of HIV or TB infection in males and females on waist circumference is not known. However, interpretation of these data is difficult because the treatment status and duration of infection for individuals was not recorded.

The present study demonstrated that male subjects treated for hypertension had a higher BMI than hypertensive males not receiving therapy. A total of 6 different pharmacological agents were being used in this population for treating hypertension, and 61.5% of hypertensive subjects were receiving 2 or more different therapies. Therefore, it was not possible to determine which specific anti-hypertensive agent was associated with the higher BMI. However, the most common agents being used were thiazide diuretics and it has been shown that hydrochlorothiazide is associated with increased levels of visceral fat [39]. Furthermore, a large proportion of hypertensive subjects were also using nifedipine, which is associated with fluid retention [40]. Further studies are required to determine why these effects on BMI were only seen in male subjects, and which anti-hypertensive agents were responsible for the association with weight gain.

Limitations of this study are that this was a cross sectional study and therefore true causation cannot be ascertained. Also, all sleep data were self-reported as per similar studies [4–6,8], and quality of sleep was not objectively assessed. A further limitation of this study is that smoking and environmental tobacco smoke exposure were not assessed via physical measurements e.g. from serum cotinine levels. Additionally, there are other variables that were not measured in this study and which may explain some of the observed associations. Thus, income was not ascertained and neither was alcohol intake or dietary consumption. Also, the number of male subjects was lower than the number of female subjects and therefore the absence of associations in males may be due to a lack of statistical power.

Data from this study suggests that sufficient sleep duration should be recommended as part of a healthy lifestyle, but more cohort studies are required to determine whether excessive sleep duration is a biological risk factor for high blood pressure and cardiovascular disease. The positive effects of daytime napping observed in the current study need to be confirmed in a larger study and more intensive investigations performed on vascular function and body fat distribution. The positive effect of anti-hypertensive agents on body weight is concerning, particulalrly in this population which has a high prevalence of hypertension. This requires further investigation to determine which specific agents are responsible and whether they have detrimental metabolic effects in this population, as has been observed with thiazide diuretics in other populations [40]. The development of comprehensive and culturally acceptable smoking-cessation interventions also requires the inclusion of weight management programmes. The dangers of environmental tobacco smoke exposure must be emphasised but further studies are required to determine the exact effects of this on body fat mass and whether avoiding passive smoke exposure can lead to weight gain.

## Supporting Information

S1 Dataset Socio-demographic data collected from primary care patients (a total of 1311 adult subjects, 862 women and 449 men) attending two pre-selected primary care clinics in Soweto, South Africa. (XLSX)

S1 Fig The effect of environmental tobacco smoke exposure on BMI in males (n = 424; grey filled bars) and females (n = 829; open bars). The data is given as median with inter-quartile range; *p<0.05, **p<0.01, ***p<0.001 versus none; ^+^p<0.05 versus 1–6 times/week.
(TIF)

S2 Fig The effect of night time sleep duration on BMI in females < 40 years-of-age (n = 414; light grey filled bars) and females ≥ 40 years-of-age (n = 409; dark grey filled bars). The data is given as median with inter-quartile range; *p<0.05 versus <8 hours.
(TIF)
