# Supplementary figures and images for: Is There an Association between Sleeping Patterns and Other Environmental Factors with Obesity and Blood Pressure in an Urban African Population?
Source: PLoS One. 2015 Oct 8;10(10):e0131081. doi: 10.1371/journal.pone.0131081 (PMC4598123; doi:10.1371/journal.pone.0131081)

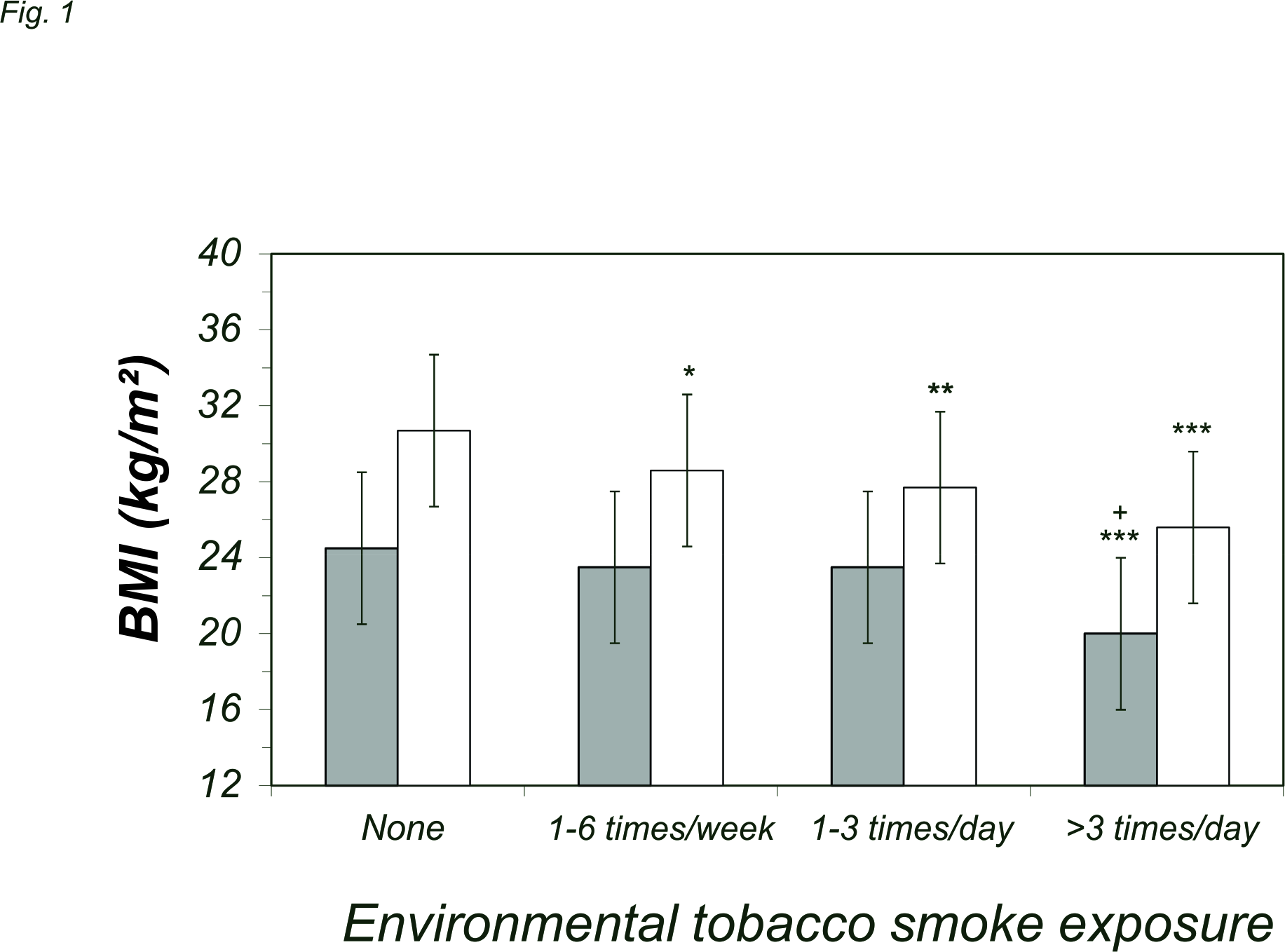

Supplement: S1 Fig — The data is given as median with inter-quartile range; *p<0.05, **p<0.01, ***p<0.001 versus none; +p<0.05 versus 1–6 times/week. (TIF) [file pone.0131081.s002.tif]

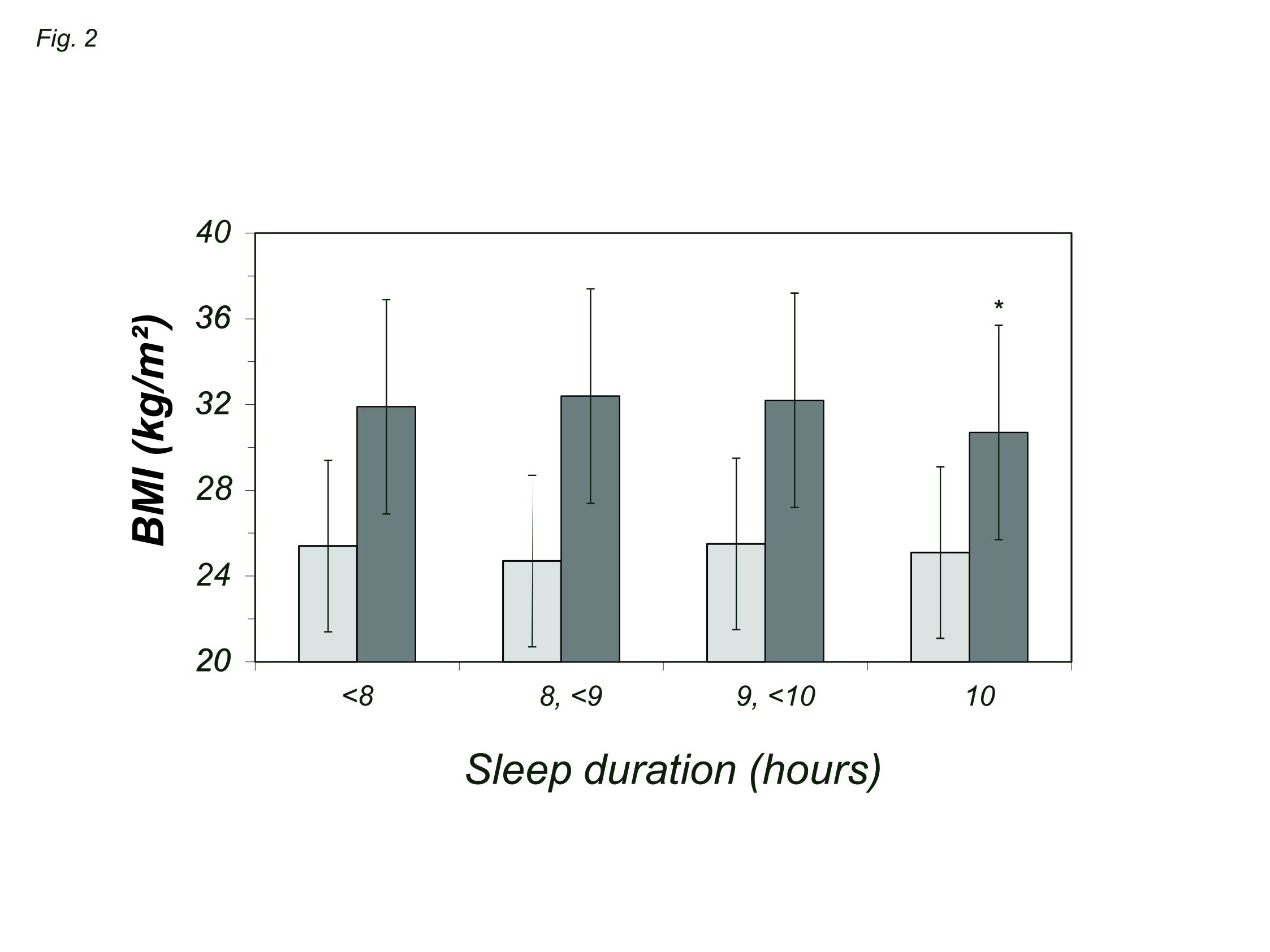

Supplement: S2 Fig — The data is given as median with inter-quartile range; *p<0.05 versus <8 hours. (TIF) [file pone.0131081.s003.tif]
